# Innovative ceramic-matrix composite substrates with tunable electrical conductivity for high-power applications

**DOI:** 10.1080/14686996.2022.2137695

**Published:** 2022-11-07

**Authors:** Driss Kenfaui, Zarel Valdez-Nava, Lionel Laudebat, Marie-Laure Locatelli, Céline Combettes, Vincent Bley, Sorin Dinculescu, Christophe Tenailleau, Pascal Dufour, Sophie Guillemet-Fritsch

**Affiliations:** a LAPLACE, Université de Toulouse, CNRS, INPT, UPS, Toulouse, France; b CIRIMAT (Centre Inter-universitaire de Recherche et d’Ingénierie des Matériaux), Université́ de Toulouse, CNRS, INPT, UPS, Toulouse, France; c Institut National Universitaire Champollion, Université de Toulouse, Place de Verdun, Albi, France

**Keywords:** Power module, ceramic-matrix composite substrate, graphene, spark plasma sintering, electrical conductivity anisotropy, breakdown voltage

## Abstract

A wide band gap semiconductor power module can operate at higher voltages as compared with its traditional silicon counterpart. However, its insulating system undergoes stronger electric fields at the triple point between the ceramic substrate, the metallic tracks and the encapsulating polymer, which can dramatically reduce its lifespan. Here we report an original concept based on the local modification of the substrate properties to mitigate such electrical stress. Numerical simulations revealed its potential to reduce this constraint by up to 50%. This concept was realized by developing, through a practical approach, a novel substrate made of an AlN-based ceramic (material A) integrating a nanocomposite volume endowed with controlled properties and geometry. This approach implies first the spark plasma sintering of the AlN powder with additives (Y_2_O_3_, CaF_2_) to endow the material A with a very low electrical conductivity (σ) and high thermal conductivity (k). Graphene nanoplatelets (GNP) were incorporated within this material to fabricate a nanocomposite with a controlled σ anisotropy that otherwise reached a striking ratio of 10^6^ at 20°C for 1.25 vol% GNP. Our approach secondly aimed at developing an effective process allowing to integrate this nanocomposite into the material A with a very high degree of reproducibility. It finally consisted in establishing the electrical contacts on the achieved substrate and encapsulating it for breakdown testing. The novel substrate enabled a mitigation of the electrical constraint by diminishing its intensity and shifting it from the triple point to a less constrained area. It already brought an improvement in breakdown voltage (V_B_) by 15% as compared to the traditional substrate, and revealed the potential for achieving higher V_B_ as well. This work lays the foundation for the development of novel multifunctional ceramic-matrix composite substrates sought for power electronics as well as for other potential applications.

## GRAPHICAL ABSTRACT

**Figure:**
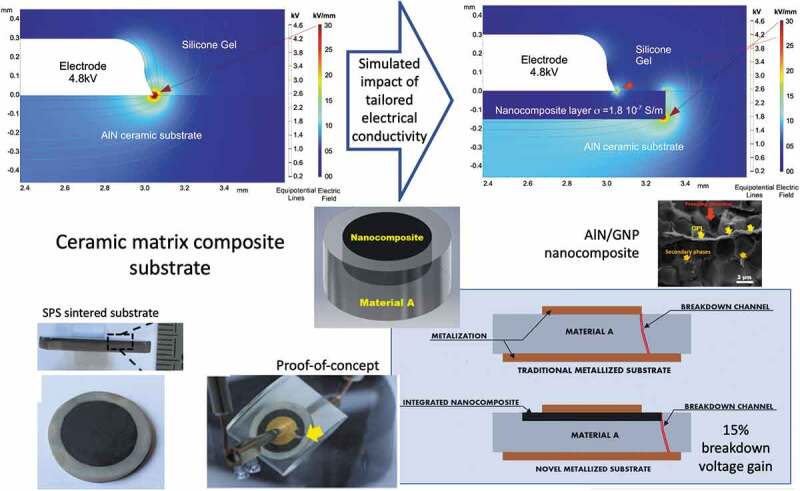

## 1. Introduction

With the unquenchable thirst of societies worldwide for energy and the emerging ecological awareness, a pressing need exists for prioritizing the use of electrical energy that represented a share of 19% of the total final energy consumption in 2018, and it is expected to grow up to 24% within the next two decades [1]. An ever-growing share of such energy is managed by the power electronics (PE), a key technology for the efficient conversion, conditioning, and control of the flow of this energy from the source to the load. This technology is associated with High Voltage Direct Current (HVDC) networks by the mean of power converters to tap into the various renewable energy sources located far from consumption centers.

**Figure 1.**
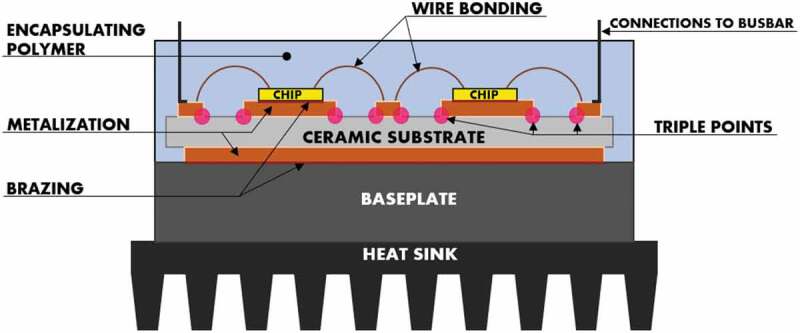
Schematic illustrating a typical representation of the traditional power module. Triple points, localized at the junction between the ceramic substrate, the metallic track borders and the encapsulating polymer, are highlighted.

A PE converter uses one (or more) power module(s) (Figure 1) which comprises semiconductor chips surrounded by an assembly of heterogeneous materials including a metallized ceramic (alumina (Al_2_O_3_), aluminum nitride (AlN) or silicon nitride (Si_3_N_4_)) [2] serving as a substrate to concomitantly ensure multiple technological functions that are essential to the operation of this device. The ceramic substrate indeed performs the dual function of electrically isolating the chips, that are brazed to it through the metallic tracks, and of extracting the heat generated by them. It also fulfils the role of a mechanical stand assuring the rigidity of the module assembly. The metallization is otherwise performed by a metal layer adhering to the substrate by using either an eutectic bonding process, such as DCB (Direct Copper Bonded) [3] or DAB (Direct Aluminum Bonded) [4], or AMB (Active Metal Brazing) one [2,5]. The connections on the top of the chips are established by the mean of bonding wires, ribbons [6] or pins [7], to electrically relate them to the metallic tracks.

Prior to the closing of the power module housing, this material arrangement is encapsulated by an insulating polymer, such as silicone gel, to achieve both the protection of chips and connections from the environment effects (humidity, vibration, and dust), and the dielectric protection along the substrate surfaces by avoiding, at high voltage, the risks of either corona effect or dielectric breakdown that could come about by surface creepage or bypassing in air.

The power modules, which are currently in service, include chips predominantly based on silicon (Si), and they cannot then be used for very high voltage and/or high-temperature applications, owing to the limited physical properties of Si [8]. The voltage level for commercial modules comprising Si chips (Silicon Insulated Gate Bipolar Transistor (IGBT) module), indeed, does not exceed 6.5 kV, and operate at junction temperatures limited between 125 and 150°C. But, the emergence of wideband gap semiconducting components, such as silicon carbide (SiC) [8,9], has enabled developing chips that would distinctly push these limits, opening hence the PE technology to new potential applications operating at higher voltage levels (up to several tens of kV), at higher temperatures (>200°C) and/or along with higher switching frequencies (several tens of kHz). Thus, SiC chips, which are capable of withstanding voltages higher than 15 kV, have already been reported [10–12], reflecting then an enormous potential for boosting the performances of power modules.

Nevertheless, such PE technological breakthrough will be accompanied by the need to miniaturize electrical systems, which will not only make it more complicated to manage the power density and extract the heat dissipated by the chips, but will also markedly intensify undue stresses within the power module. Effectively, increasing the voltage level in the current power module results in heightening the electric field at the junction between the ceramic substrate (Figure 1), the metallic track borders and the encapsulating polymer [13]. The induced electrical constraint at this triple point can dramatically impair the performances of the power module and, subsequently, reduce its lifespan, when it exceeds the critical one allowed by the dielectric strength of the insulating materials, thereby limiting the voltage ratings for future systems. Note that an increase in electric field within the power module may also lead to a premature electrical aging of the insulating materials, even when operating at voltage levels below their breakdown thresholds. Such material aging is mainly triggered by the occurrence of a localized activity of partial discharges that occur in microcavities that may be present within the insulating volume, at the outer edge of the metallization and, in particular, at the above-mentioned triple point [14–16].

**Figure 2.**
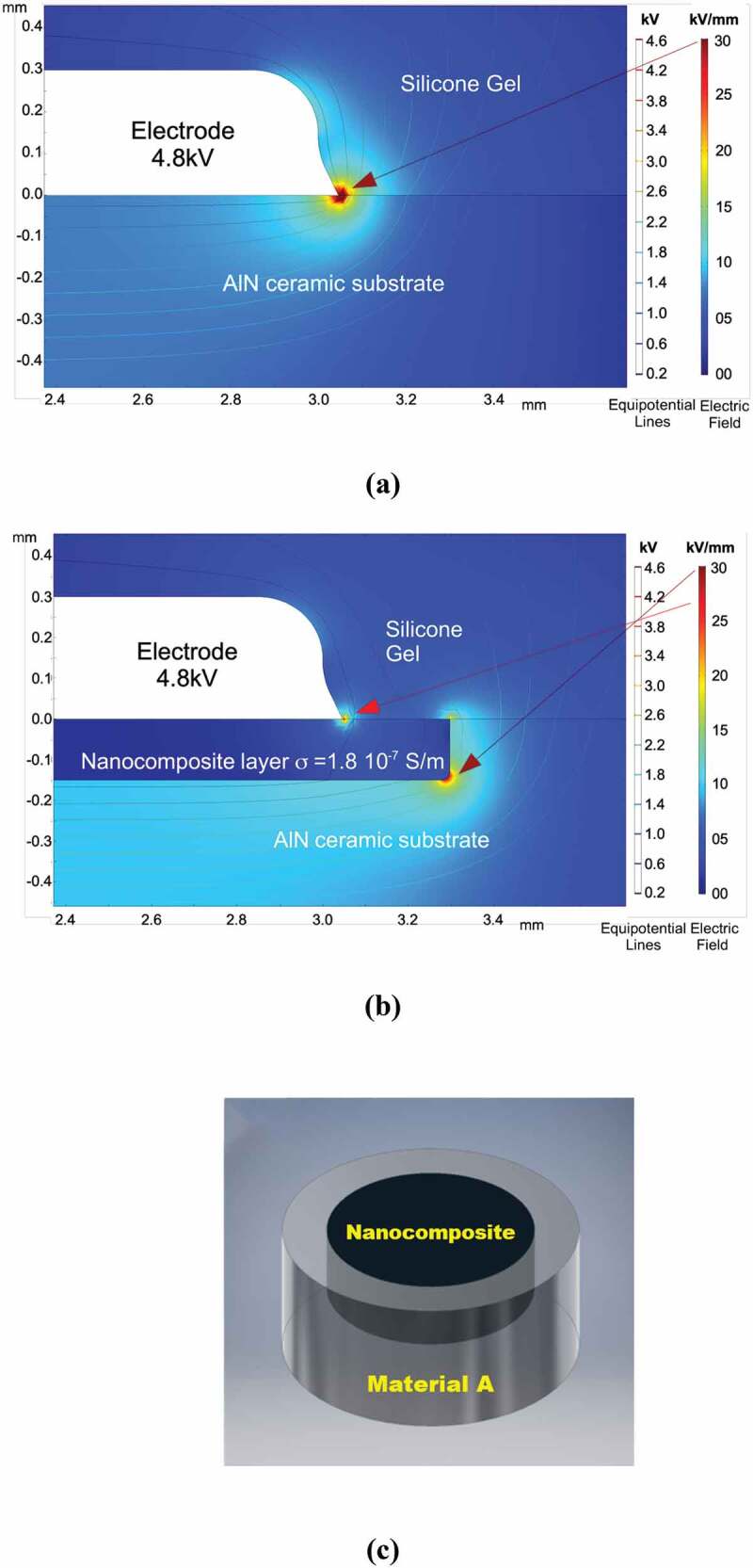
FEM simulation of power modules comprising **(a)** the traditional substrate which highlights the concentration of the electrical field stress around the triple point area, and **(b)** the novel substrate made of AlN ceramic (10 ^−13^ S.m ^−1^) integrating a three-dimensional material zone with a 150 µm in depth, possessing a larger electrical conductivity (~10 ^−7^ S.m ^−1^). the maximum electrical field in the novel substrate is reduced around 50% compared to the traditional AlN one. **(c)** Schematic illustrating the novel substrate made of AlN-based ceramic (Material A) integrating a material volume including a nanocomposite endowed with controlled properties and geometry.

Accordingly, we beforehand undertook a preliminary modeling investigation, based on the finite element simulation of the electric field distribution within a power module, to evidence such electrical constraint in typical geometry. The power module comprises a 635 µm – thick AlN ceramic substrate with a dielectric permittivity, ε_AlN_ = 8.8, and an electrical conductivity, σ_AlN_ = 10^−15^ S.cm^−1^. This module is encapsulated by a silicone gel (ε_gel_ = 2.7 and σ_gel_ = 10^−17^ S.cm^−1^). An alternative voltage of 4.8 kV at 50 Hz is applied on one 300 µm thick – copper electrode on the upper side of the substrate, whereas the other electrodes on the upper and back sides are linked to the ground. The achieved numerical results are depicted in Figure 2a which indeed shows a concentration of equipotential lines around the triple point within the addressed module, resulting in heightened electric field stresses, located in the ceramic substrate and the gel, with estimated maximum values of 56.9 and 50.4 kV mm^−1^, respectively.

Most of the solution strategies published to date for mitigating this electrical stress involve polymer encapsulation. Yet, it has been reported that in a power module, the activity of partial discharges seems to originate from the ceramic substrate, rather than from the encapsulating material [17], and the gel possesses a higher dielectric strength and appears to undergo less electrical stress compared to the ceramic substrate as highlighted in Figure 2a. These strategies mainly involve modifying the encapsulation polymer *i)* by adding micro- and/or nanoparticles to improve the dielectric properties (breakdown or partial discharges thresholds) [15], and *ii)* by using anisotropic materials or properties gradient materials as functionally graded materials to allow a self-adaptive permittivity gradient at the area where the constraints are more prominent [18,19.

Here, we address that issue of electrical field heightening by acting, for the first time to our knowledge, on the insulating ceramic substrate. We report therefore an original concept based on locally amending its properties with the aim of attenuating the reinforced electrical constraint concentrated at the triple point by shifting it, as well as reducing its intensity, towards an area that is otherwise less constrained within the power module. This concept was realized by developing, through a practical approach, a novel substrate made of an AlN-based ceramic (material A) integrating, immediately underneath the metallization, a nanocomposite volume with controlled properties and geometry. The chemical composition and the microstructure features of both material A and nanocomposite were adjusted using the required additives and the Spark Plasma Sintering (SPS) to achieve the properties suited for endowing the novel substrate with the multiple technological functions including the electrical field grading sought for a high-power module. An effective process was then developed to three-dimensionally integrate the nanocomposite within the material A. The achieved novel substrate was finally used to fabricate a power-module packaging prototype that was experimentally assessed in terms of the dielectric strength. The experimental results were compared with the simulation predictions, which enables highlighting the merits of such concept and validating it.

Paragraph: use this for the first paragraph in a section, or to continue after an extract.

## 2. Experimental section

### 2.1. Powder mixture preparation

*T*he starting powder AlN (median grain size d_50_ = 1.9 µm, Atochem, France) and the sintering additives Y_2_O_3_ (purity: 99.9%, Shinetsu, Japan) and/or CaF_2_ (purity: 99.7%, Alfa Aesar, Germany) were weighed in the proper stoichiometric ratios and mixed at 200 rpm for 1 h in an agate ball mill using absolute ethanol as a dispersant. The obtained mixture was then dried in air at 80°C for 15 h to eliminate this liquid.

### 2.2. Exfoliation of graphite

A liquid solution of 2.5 vol.% of graphite nanopowder in 20 ml of isopropyl alcohol was hence thoroughly prepared and transferred to the probe-type sonicator (Sonics Materials VCX-750, Fisher Scientific, France), fitted with a 13 mm circular tip and a high-frequency ultrasonic generator. In all the experiments, the sonicator power was set at 150 W with an amplitude of 40% and a pulse mode defined by the cycle of 4s ON/6s OFF. The pause time (6s OFF) enables limiting the temperature rise of the solution, thus slowing the evaporation of the solvent. Note that the latter was added along the sonication to maintain the solution volume constant.

### 2.3. SPS processing

For each sample, 2 g of the prepared powders (AlN +3 wt% Y_2_O_3_, AlN +1 wt% Y_2_O_3_ +2 wt% CaF_2_ or AlN +1 wt% Y_2_O_3_ +2 wt% CaF_2_ +2.5 vol.% GNP (graphene nanoplatelets)) were loaded in a graphite die with an inner diameter of 20 mm which was then transferred to the SPS machine and heated up to the dwell temperature of 1800°C for 10 min, with a heating rate as high as 100 °C/min, and under pressure level of 50 MPa. Note that an AlN sample was elaborated from pure AlN powder under the same SPS conditions and according to the procedure described above. The as-prepared samples were then polished to remove the graphite foil used during SPS processing before undergoing further characterizations.

### 2.4. TEM characterization

To characterize the exfoliation state, a similar solution was sonicated for 9 h and, immediately after the end of the sonication, a few solution drops, loaded with exfoliated graphite, were deposited on holey carbon grids for observation by transmission electron microscopy (TEM). After evaporating the isopropyl alcohol, the structural and morphological characterizations of the exfoliated graphite were performed on a TEM (HT-7700 Hitachi 120 kV) operated at an accelerating electron voltage of 80 kV to diminish the damage that can stem from the electron beam on the graphene while maintaining the image resolution of the particle fringes in attempt to distinguish the number of layers and/or the local thickness on folded edges.

### 2.5. Dielectric spectroscopy

The dielectric properties were monitored for the materials under low voltage (3 Vpp) in the 10^−1^-10^6^ Hz frequency range by using a broadband impedance analyzer (Novocontrol Alpha-A). The elaborated pellets of 20 mm in diameter were mirror-polished before being annealed at 150°C during 5 h to eliminate all traces of water. A 80 nm-thick gold electrode was deposited by sputtering on both faces of each sample. The metalized pellet placed into the measuring cell between two conducting electrodes to form a parallel capacitor. Alternative voltage, U(t) = U_0_ cos(t) was applied to the latter at a controlled frequency. A subsequent electrical current was generated with a difference in amplitude and phase shift I(t) = I_0_ cos(ωt+ϕ). The complex impedance is given by

$$Z^{*}\left(\omega \right)=Z{\;}^{\prime }\left(\omega \right)+jZ\left(\omega \right)=U^{*}\left(\omega \right)/I^{*}\left(\omega \right)$$

where Z*(ω) stands for the Fourier transform of the complex impedance and ω = 2 π/f (rad/s) is the angular frequency. U*(ω) and I*(ω) are the complex notations of U(t) and I(t), respectively. Zʹ(ω) and Zʺ(ω) represent the respective real and imaginary parts of the impedance, in linear case; the ratio U*(ω)/I*(ω) is independent of the magnitude U_0_.

The complex permittivity ε* and conductivity σ* are deduced from the impedance as follows:

$$\varepsilon^{*}=\varepsilon^{\prime }-i\varepsilon =\left(-i\right)/(\omega \;x{\;Z}^{*}(\omega )\;x{\;C}_{0})$$

$$\sigma^{*}=\sigma^{\prime }-i\sigma "=d/(Z^{*}\left(\omega )\;x\;A\right)$$

where C_0_ (F), d (m) and A (m^2^) represent the capacitance of empty cell and the electrode spacing and area, respectively. (εʹ; εʺ) and (σ ʹ [S/m]; σʺ [S/m]) are the respective real and imaginary parts of the permittivity and conductivity.

### 2.6. Thermal measurements

The thermal conductivity κ was determined in the 50–400°C temperature range as follows: κ = d × C_p_ × D, where d and C_p_ are the density and the specific heat capacity, respectively, while D is the thermal diffusivity. D was measured by the laser flash diffusivity method by using the LFA 457 MicroFlashTM system (NETZSCH-Gerätebau GmbH, Selb/Germany) on ∼6 mm × 6 mm × 2 mm parallelepiped-shaped specimens cut from the core of 20 mm diameter – pellets. C_p_ and d were determined by using the thermal analyzer (STA 449 F3 Jupiter®, NETZSCH-Gerätebau GmbH, Selb/Germany) and Archimedes method (KERN & Sohn GmbH, Baligen, Germany), respectively.

## 3. Modelling section

The finite element method (FEM) was used to simulate the electric field distribution within *i)* the first power module with a traditional AlN substrate (Figure 2a) and *ii)* the second one comprising the novel AlN substrate integrating the tridimensional zone, that is electrically more conductive (Figure 2b), in effort to ascertain the effect of this latter and to evaluate such substrate concept. For both cases, the simulation offers a quantitative assessment of the resulting electric field.

**Figure 3.**
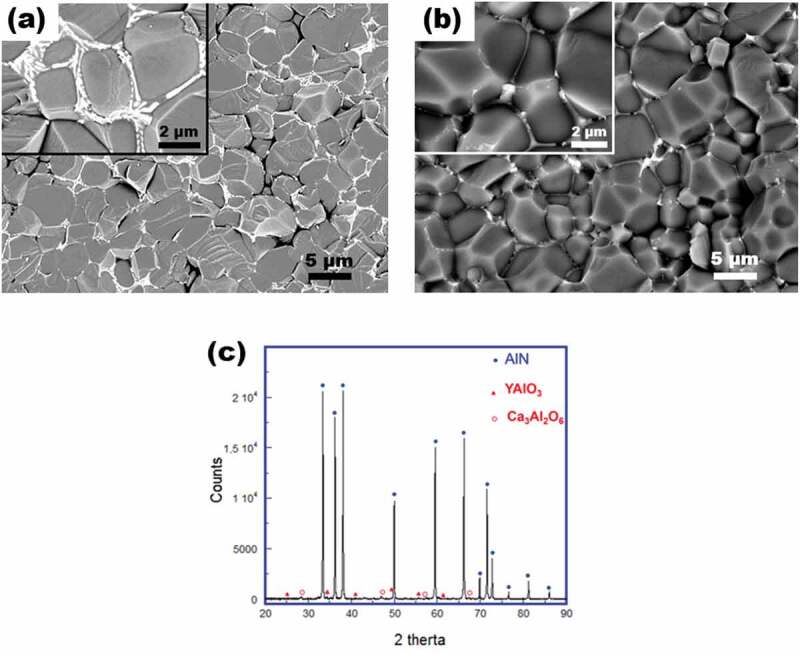
SEM micrograph of fractured surface parallel to the applied axis of the (a) AlN +3 wt% Y_2_O_3_ and (b) AlN +1 wt% Y_2_O_3_ + 2 wt % CaF_2_ ceramic samples processed by SPS in N_2_ atmosphere at 1800°C under 50 MPa for 10 min. (c) X diffractogram recorded for the AlN +1 wt% Y_2_O_3_ + 2 wt % CaF_2_ sample (material A) indicating the formation of the oxides Ca_3_Al_2_O_6_ and YAlO_3_ during the SPS sintering.

To achieve that, an electrostatic analysis based on Maxwell’s equations was undertaken by solving the equations below to compute electric field, current and potential distributions in conducting media under conditions where inductive effects are negligible:

$$\vec{E}=-\vec{\nabla }V$$

$$\vec{D}=\varepsilon_{0}\varepsilon_{r}\vec{E}$$

$$\vec{J}=\sigma \vec{E}+\frac{\partial \vec{D}}{\partial t}$$

where ε_0_ = 8.85 × 10 ^−12^ F m ^−1^ refers to the vacuum dielectric constant.

The 2D problem is solved using Comsol Multiphysics 5.3a computing in a frequency domain. The maximum magnitude of the electric field is estimated for a sinusoidal voltage 4.8 kV at 50 Hz applied on one copper electrode (the other electrodes are related to the ground). The presence of electric field singularities (corners, triple points, tips, etc.) is a problem from the numerical point of view. Indeed, the mesh is dependent on the electric field strength and a numerical solution will depend on the mesh resolution as reported elsewhere [20] (the theoretical value of the field tends to infinity). Hence, the electric field was simulated using the same mesh for both module cases (Figures 2 a and b). Here, the structure is defined using a fixed triangular mesh (30 um).

To address the electrical insulation system, the properties as well as the dimensional parameters of the traditional and novel substrates, the encapsulating silicone gel and the copper electrodes were used for simulation. A 650 µm – thick ceramic metallized with electrochemically etched electrodes and encapsulated in silicone gel allows us to obtain the geometry presented in Figure 2, that is too often used in power modules. The material parameters, considered here, are the permittivity and the electrical conductivity that are equal, respectively, to 2.7 and 10^−17^ S.cm^−1^ for silicone gel, 8.8 and 10^−15^ S.cm^−1^ for ceramic and 8.8 and 1.8 10^−9^ S.cm^−1^ for the nanocomposite layer. The maximum magnitude of the electric field is then evaluated for each configuration and presented in Figures 2 a and b.

## 4. Assessment of the concept by finite element simulations

Before realizing this concept, we assessed it by performing numerical investigations by finite elements for the same module, but, this time, it comprises furthermore an AlN ceramic substrate integrating a three-dimensional material zone with 150 µm in depth, possessing a higher electrical conductivity (~10^−9^ S.cm^−1^), located immediately underneath the metallization (Figure 2b). The simulation results exhibited that the use of such novel substrate brought a drastic reduction in the electrical constraint at the triple point through the spreading of equipotential lines caused by the more conductive zone. Indeed, for the same sinusoidal voltage supply of 4.8 kV at 50 Hz, the maximum electrical field in the substrate is decreased to 30 kV/mm compared to 56.8 kV/mm previously noted in the traditional AlN substrate (Figure 2a), corresponding to a decrease of close to 50%. The electrical constraint was diminished and shifted to the edge of the integrated zone, where the electrical fields in the gel (near the substrate surface) and in the more conductive zone are estimated at 24.7 and 27 kV/mm, respectively, as well.

## 5. Design and development of the novel substrate for electrical field grading

To realize the concept, we developed a practical approach aiming to fabricate a novel substrate made of AlN-based ceramic (material A) integrating a volume including a nanocomposite endowed with controlled properties and geometry as schematized in Figure 2c. The nanocomposite was developed by partially modifying the chemical composition of the material A through the incorporation of carbon-based nanoparticles. Note that AlN material was selected here as the ceramic matrix because of its better properties for power modules as compared to the Al_2_O_3_ and Si_3_N_4_ ones [2]. Indeed, it possesses both a high dielectric strength and a very low electrical conductivity, less than 10^−15^ S⋅cm^−1^ at room temperature, characteristics of an excellent dielectric material well-suited for withstanding higher voltage level and electrically isolating the chips in the module power. It exhibits a much larger thermal conductivity as well, making it more relevant for dissipating the heat generated by them.

Even so, elaborating AlN ceramics by conventional sintering requires high temperature levels combined to very long times to reach a high material density, owing to the strong covalent bonds in the AlN system and the low diffusion coefficient of its constituent elements [21–23]. Long sintering times will not only push up the cost of fabrication of these materials, but will also promote the growth of their grains and, subsequently, cause the degradation of their mechanical properties.

We hence used the SPS process, well-known for its ability to speed up the sintering kinetics and to achieve very high material density [24], to elaborate the AlN-based samples (material A and nanocomposite) needed for experimental investigations, and, in particular, to integrate the nanocomposite within the material A to fabricate the novel substrate.

### 5.1. Fabrication of fully densified AlN-based ceramics (material A)

In order for the novel substrate to fulfill the aforesaid technological functions concomitantly with its role of grading the electrical field, the material A must possess, besides a very low electrical conductivity, the highest possible thermal conductivity. This material must hence display a full densification, which is otherwise needed to limit the local activity of partial discharges within its microstructure pores at high voltage [17], on the one hand, and to ensure good mechanical properties, on the other hand.

For these purposes, we added, prior to SPS processing, a fraction of 3 wt% Y_2_O_3_ to the AlN powder, as this sintering additive was reported to react with the latter to form a liquid phase allowing to foster densification through liquid-phase sintering [22–24], and to reduce the oxide impurities in the AlN lattice that have proved probative for the thermal conduction [25]. The AlN +3 wt% Y_2_O_3_ mixture was prepared as per the procedure described in the methods’ section (*Powder mixture preparation*), and treated thereafter in nitrogen atmosphere in the SPS apparatus (Dr. Sinter 2080 device from Sumitomo Coal Mining – Fuji Electronic Industrial, Saitama, Japan) under the conditions and following the procedure detailed in this section (*SPS processing*) as well.

Scanning electron microscopy (SEM) observations, performed on a fractured surface obtained by cleavage parallel to the applied pressing axis of the AlN +3 wt% Y_2_O_3_ pellet (Figure 3a), showed the typical microstructure features of AlN ceramics produced by SPS with sintering additives, *i.e*., the secondary phases (in bright contrast) are placed at the grain boundaries of the AlN matrix. Such microstructure is nearly fully densified (>98%) with a very limited grain growth. It was achieved by the fact that the sintering occurred in the liquid phase formed following the reaction of Y_2_O_3_ with the thin Al_2_O_3_ layer originally present on the surface of the AlN grains. The secondary phase, in a liquid form, flowed into the grain boundaries before being solidified during cooling, thereby densifying the material. Its chemical composition was found to be close to that of YAlO_3_, which is in keeping with that reported elsewhere [25]. Nonetheless, it has to be noted here that adding a fraction of Y_2_O_3_ as high as 3 wt% led to the formation of a relatively large amount of YAlO_3_ which partly covers the AlN grains as can be clearly seen in the inset of Figure 3a. That would likely promote the diffusion of phonons at the YAlO_3_ /AlN interfaces, and subsequently degrade the thermal conduction within the material.

To cope with such YAlO_3_ excess, we decreased the Y_2_O_3_ fraction to 1 wt% and added 2 wt% of CaF_2_, as the latter is reported to lead to the formation of compounds that evaporate during sintering [23], thus reducing the amount of secondary phases at the grain boundaries. The resulted AlN +1 wt% Y_2_O_3_ +2 wt% CaF_2_ mixture was homogenized by using the same procedure given in the methods’ section, and consolidated then by SPS under analogous conditions.

Figure 3b depicts the SEM micrograph of a fractured surface of the AlN +1 wt% Y_2_O_3_ +2 wt% CaF_2_ sample elaborated by SPS. It depicts a fully densified (>99%) and homogeneous microstructure containing a much lower amount of secondary phases sited at the junction lines and concentrated exactly at the triple points between the grains (Insert of Figure 3b). It emphasizes therefore the relevance of the selected experimental conditions as well as the choice of the sintering additives and the adjustment of their fraction. This is mainly ascribed to the formation of some secondary phases that evaporated during the sintering. Indeed, traces of evaporated compounds were observed on the spacers at the end of the SPS processing. The XRD patterns show the presence of an additional oxide, Ca_3_Al_2_O_6_, coexisting with YAlO_3_ at the grain boundaries (Figure 3c). The production of the compounds AlF_3_, Ca_3_N_2_ and Ca_11_N_8_ has been reported [23]. Their decomposition temperature [23] is lower than the dwell one (1800°C) used here, thereby they readily evaporate during the sintering.

In light of these results, the AlN +1 wt% Y_2_O_3_ +2 wt% CaF_2_ composition was selected as the material A for fabricating the novel substrate.

### 5.2. Fabrication of nanocomposites endowed with controlled properties

As already stated above, the material volume, which will be integrated in the material A to fabricate the novel substrate, comprises a nanocomposite endowed with controlled properties. Compared to the material A, it must then possess a higher electrical conductivity with a magnitude enabling grading the electrical field, concomitant with the closest possible thermal conductivity to prevent thermal stresses in the novel substrate.

Thus, we developed a nanocomposite by incorporating the graphene nanoplatelets (GNP) into the material A in an effort to capitalize on their combination of large specific surface area, two-dimensional high ratio aspect sheet geometry and very high charge carrier mobility [26–29]. The choice of GNP is also dictated by their outstanding mechanical properties [30,31] that endow them with high resistance to irreversible damage during harsh SPS sintering at a temperature as high as 1800°C under loading required for fully densifying AlN-based materials. Such resistance distinguishes them from carbon nanotubes (NTC) that are reported to be reactive with oxides and nitride-based materials in similar environments, making it difficult for them to keep their structure upon SPS processing [32,33].

#### 5.2.1. Production of graphene platelets (GNP)

With a view of exploiting such properties of GNP, we performed a simple and cost-effective procedure to produce these nanoparticles by solution-phase exfoliation of graphite (Average lateral particle size: 15 µm, Skyspring Nanomaterials, Inc. - USA) in isopropyl alcohol using ultrasonication. This solvent was selected for its evaporation temperature (82.6°C) that is much lower than that of (NMP) *N*-methyl-2-pyrrolidone (202°C) or (DMF) *N*-dimethylformamide (153°C) solvents regularly used to stabilize the dispersion of graphene sheets prepared by exfoliation of graphite [34]. Indeed, it will be easier to eliminate during the preparation of the nanocomposite powder.

**Figure 4.**
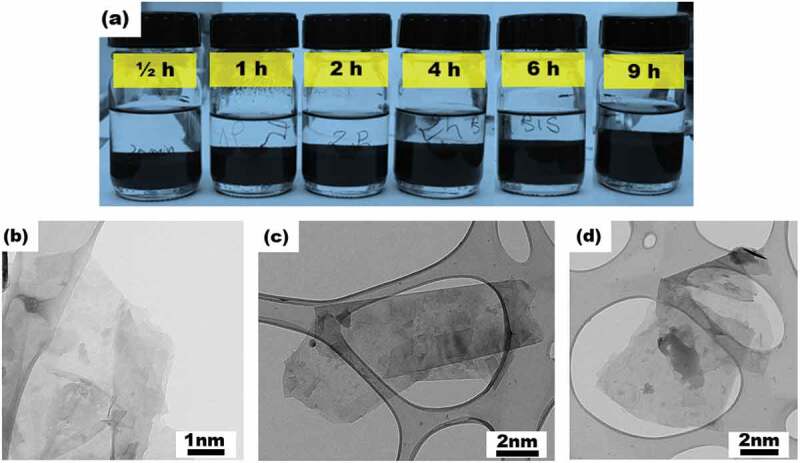
(a) Similar solution samples, containing 2.5 vol.% of graphite nanopowder in 20 ml of isopropyl alcohol, separately sonicated for different effective times (0.5, 1, 2, 4, 6 and 9 h) before being left to settle for 15 h. The apparent volume (black part of the container) of the exfoliated graphite increases with the sonication time, highlighting the increase of GNP amount. (b-d) TEM micrographs of graphene flakes deposited over the TEM grid immediately after the end of the sonication of a similar solution for 9 h. They present (b and c) two-dimensional and quasi-transparent flakes revealing nanometric thicknesses and lateral sizes in the 600–2500 nm range, and (d) less transparent and thicker flakes comprising a higher number of single-layers.

Thus, a series of similar solution samples, prepared from 2.5 vol.% of graphite nanopowder in 20 ml of isopropyl alcohol, were separately sonicated following the procedure and under the conditions given in the methods’ section (*Exfoliation of graphite*) for different times before being left to settle with the aim of evidencing the evolution of graphite exfoliation with sonication time under the selected conditions. Figure 4a shows an image taken 15 h after the solution samples were sonicated for the effective times (pulse: on) of 0.5, 1, 2, 4, 6 and 9 h. The longer the sonication time, the larger the apparent volume (black part of the container) of the exfoliated graphite, suggesting the increase of GNP amount with sonication time.

The TEM characterization of the solution sonicated for 9 h evidenced a large number of two-dimensional and quasi-transparent flakes were hence observed (Figures 4 b and c) exhibiting lateral sizes in the 600–3500 nm range, which could be comparable to those reported for single-layer graphene flakes obtained by exfoliation of graphite in the NMP solvent [34] or in other liquids [35,36]. In many cases, the graphene flakes depict straight edges and corners with angles well-suited for allowing the observation of the nanometric order of their thickness and even the very small number and presence of single-layer flakes. We also observed the existence of less transparent and thicker flakes as shown in Figure 4d, comprising a higher number of single-layers.

#### 5.2.2. Elaboration of nanocomposites

Immediately upon 9 h – sonication of a new solution sample of 2.5 vol.% of graphite in 20 ml of isopropyl alcohol, the obtained solution was introduced with the AlN +1%wt Y_2_O_3_ + 2%wt CaF_2_ powder into a glass flask that was then fixed in a rotary evaporator, and subjected to a rotation of 70 rpm for 2 h in view of homogenizing the resulting solution. It was then heated up to 70°C under vacuum while maintaining the flask rotation. The produced vapor was progressively pipped to a cooler system for condensation, thereby removing the solvent to obtain the nanocomposite powder AlN +1%wt Y_2_O_3_ + 2%wt CaF_2_ + 2.5%vol GNP (material B). This mixture was annealed at 80°C for 15 h before being treated in SPS under the same aforementioned conditions.

### 5.3. Electrical and thermal properties

**Figure 5.**
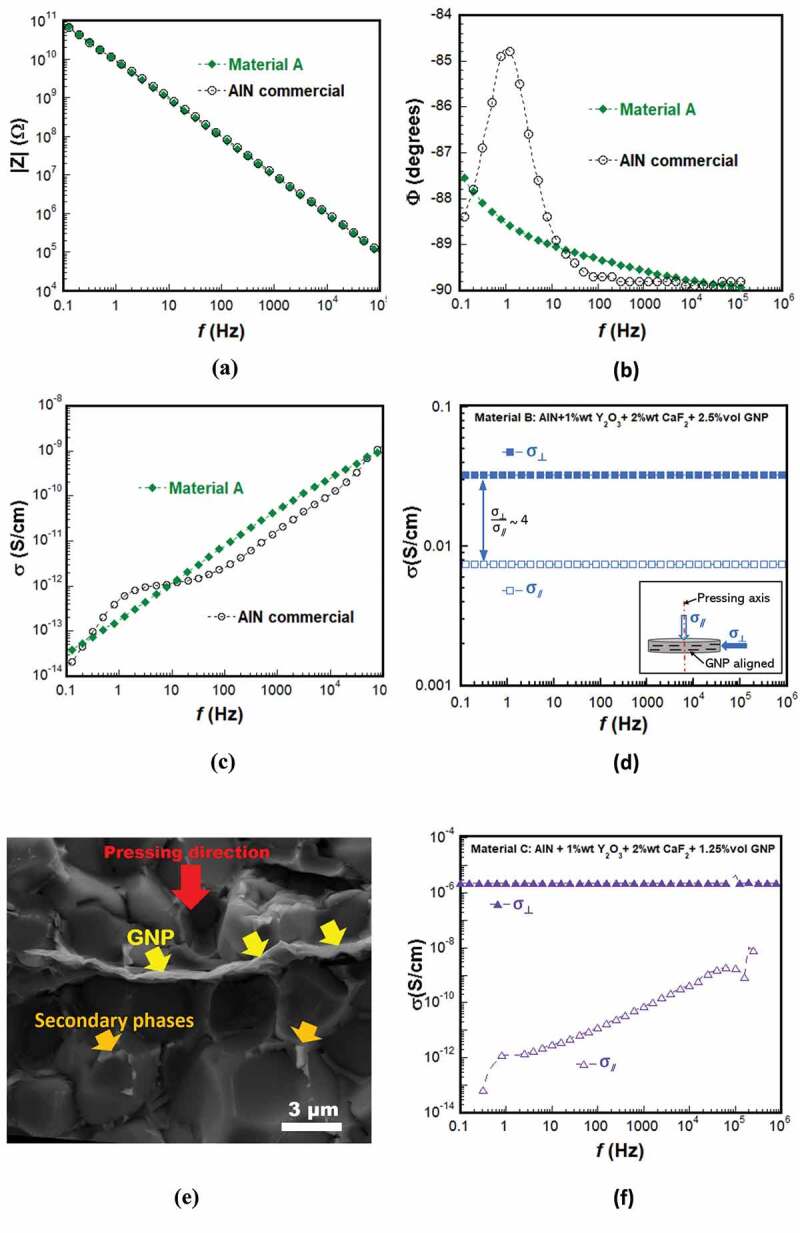
The frequency dependence of (a) the impedance ∣Z∣, (b) the phase angle ϕ and (c) the electrical conductivity σ measured for the material A at room temperature. The frequency dependence of the electrical conductivity monitored in the directions parallel (σ_*∥*_) and perpendicular (σ⊥) to the applied pressing axis for the nanocomposites: (d) material B and (f) material C. (e) SEM micrograph of a fractured surface, obtained by cleavage parallel to the applied pressing axis of the material B, evidencing a stack of GNP aligned perpendicular to this axis.

In Figure 5a-c, depicted are the frequency dependence of the dielectric properties measured for the material A at room temperature. For the sake of a comparison, commercial AlN ceramic (IMPAK company, France) was characterized as well. Note that the latter differs from the SPS-sintered AlN sample.

Compared to this commercial material, the impedance curve (∣Z∣) showed that the material A sintered by SPS (Figure 5a) exhibits a similar dielectric behavior governed by the capacitive effect over all the measuring frequency range. The material A, however, seems to display a better electrical response at low frequency as can be seen from the phase ϕ and conductivity σ curves (Figure 5b-c) where its ϕ and σ were shifted to lower values. It exhibits a σ value of ~ 3 × 10^−14^ S.cm^−1^ at 0.1 Hz.

In view of the two-dimensional character of the GNP particles, the electrical conductivity of the material B was otherwise monitored on bars cut from a SPS-sintered pellet in the directions parallel (σ_*∥*_) and perpendicular (σ${\;}_{⊥}$) to the applied pressing axis (Inset of Figure 5d). Figure 5d depicts the frequency dependence of σ_*∥*_ and σ${\;}_{⊥}$ for the material B. It reveals that, whatever the measuring direction, the addition of 2.5%vol GNP made σ insensitive to the frequency over all the measuring frequency range, resulting in electrical changing from the capacitive behavior (Figure 5c) to the resistive one (Figure 5d) and significantly increasing σ to larger values (σ${\;}_{⊥}$ = 3.2 x 10^−2^ S.cm^−1^, σ_∥_ = 0.73 x 10^−2^ S.cm^−1^ at 20°C at 0.1 Hz) as compared to the material A (Table 1). The GNP therefore induced an anisotropy within the material B yielding a ratio (σ${\;}_{⊥}$ /σ_*∥*_) = 4.4, which can be derived from the fact that they were aligned, under the effect of uniaxial pressure during the SPS sintering, in the direction perpendicular to the pressing axis as can be revealed by the SEM observations (Figure 5e). Effectively, since GNP are typified by the superior σ in their in-planes over that of their out-planes, they induce σ${\;}_{⊥}$ larger than σ_*∥*_ within the material B. Note that only stacks comprising a high number of GNP could be visible using SEM. The single graphene flacks, that are also incorporated in the AlN matrix, could not be observed, but they do contribute markedly to the electrical conduction within this material.

**Table 1.** The chemical composition of the material A and the nanocomposites (materials B and C), and their electrical conductivity σ obtained at 20°C. For the materials B and C, given are the σ values recorded in the directions parallel (σ∥) and perpendicular (σ${\;}_{⊥}$) to their applied pressing axis, and their respective anisotropy (σ${\;}_{⊥}$/σ∥) ratio.

	Chemical composition	σ (S/cm)	Anisotropy ratio σ${\;}_{⊥}$ /σ_*∥*_
Material A	AlN +1 wt% Y_2_O_3_ +2 wt% CaF_2_	10^−14^	-
Material B	AlN +1 wt% Y_2_O_3_ +2 wt% CaF_2_ +2.5 vol.% GNP	σ${\;}_{⊥}$ = 3.2 x 10^−2^ σ_∥_ = 0.73 x 10^−2^	4.4
Material C	AlN +1 wt% Y_2_O_3_ +2 wt% CaF_2_ +1.25 vol.% GNP	σ${\;}_{⊥}$ = 2 × 10^−6^ 10^−14^ < σ_∥_ < 10^−13^	~10^−6^ (at low frequency)

It is relevant to point out here that such anisotropy could be advantageously used to bestow the novel substrate with the role of grading the electrical field whilst keeping the traditional functions. For that purpose, the σ${\;}_{⊥}$ electrical conductivity of the nanocomposite, that is probed here in the direction parallel to the upper surface of the substrate (Figure 2a)), must be lower than that value recorded in the material B in order to foster the electrical field grading as indicated by the simulation results. Moreover, the σ_*∥*_ conductivity (in the direction perpendicular to the surface of the substrate) should even be comparable to that of the material A so as to safeguard the function of the electrical isolation even along a depth as small as that of the thickness of the integrated nanocomposite.

To cope with this challenge, we elaborated, under similar conditions, a second nanocomposite comprising, this time, a lower fraction of GNP, *i.e*., AlN +1%wt Y_2_O_3_ +2%wt CaF_2_ +1.25%vol GNP (material C). Unlike the material B, the material C was found to present distinct electrical behaviors flowing its both main directions (Figure 5f). Indeed, it exhibits capacitive and resistive behaviors in the directions parallel (σ_*∥*_) and perpendicular (σ${\;}_{⊥}$) to the applied pressing axis, respectively, with a σ${\;}_{⊥}$ = 2 × 10^−6^ S.cm^−1^ at 20°C and σ_*∥*_ is comparable to that of the material A (Table 1).

The material C particularly reveals much more pronounced σ anisotropy with a striking (σ${\;}_{⊥}$ /σ_*∥*_) ratio attaining 10^6^ at low frequency, which is the highest value achieved hitherto for the AlN-based ceramics to our knowledge.

These results reveal that the electrical conductivity σ${\;}_{⊥}$ exceeds by far the percolation threshold (as in the case of the material B following its both main directions), whereas the σ_*∥*_ one appears to be still close to it. They otherwise suggest that, in the material C, the GNPs seem to be better aligned compared to the material B, which led to much lower σ_*∥*_ (Table 1). In other words, an important number of GNP contained in the material B, which could not be visible through SEM observations, were not well-aligned perpendicular to the pressing axis during the SPS processing, and contribute then in the electrical conduction following the direction parallel to the latter as well, thereby enhancing σ_*∥*_ and, subsequently, yielding lower anisotropy ratio. This would ultimately indicate that the lower GNP fraction led to better GNP orientation in the nanocomposite during the SPS processing.

**Figure 6.**
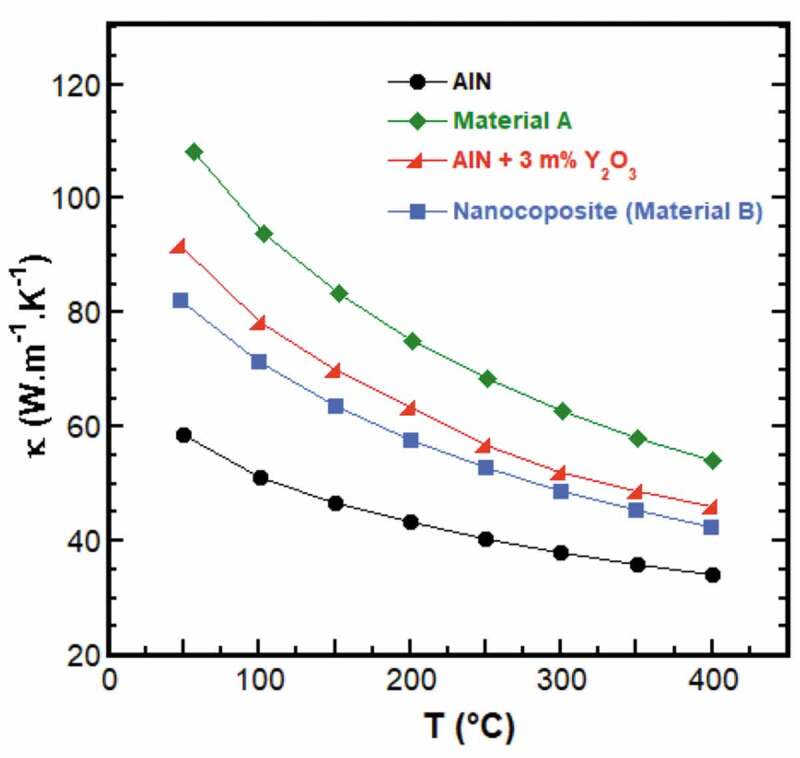
Temperature dependence of the thermal conductivity κ measured in the 50–400°C range for the material A and B. To ease a comparison, the κ-curves obtained for pure AlN and AlN +3%wt Y_2_O_3_ samples are plotted as well.

Figure 6 shows the thermal conductivity, κ, measured in the 50–400°C range for the materials A and B. In order to ascertain the effect of the selected sintering additives, the κ-curves obtained for AlN and AlN +3%wt Y_2_O_3_ SPS-sintered samples, processed under the same conditions compared to the materials A and B, were plotted as well. Generally, κ decreases as the temperature is raised, and its magnitude evolution from one sample to another mainly depends on the secondary phases as all the elaborated materials were fully densified. Effectively, adding 3%wt Y_2_O_3_ allowed to markedly increase κ because it diminishes the O_2_ impurities primarily existing in AlN material through the formation of YAlO_3_ as previously noted. But here, the YAlO_3_ was produced in abundance and then partially covers the AlN grains (Figure 3a), which consequently promotes the phonon diffusion at the AlN/YAlO_3_ interfaces. It results in diminishing the mean free path of the phonons which is proportional to the lattice component of κ that is dominant in the AlN material, thereby limiting κ.

Such phonon diffusion was lowered for the material A by simultaneously decreasing the Y_2_O_3_ fraction and adding CaF_2_. Indeed, that brings a significant reduction in the number of AlN/secondary phases through the formation of evaporable secondary phases during the sintering (Figure 3b), hence rising κ in the material A. Note that κ improvement is achieved by concomitantly controlling the O_2_ impurities and the interfaces AlN/secondary phases in the material A.

Otherwise, incorporating GNP in the material A led to a decrease in κ (in the material B), which can be obviously ascribed to the phonon diffusion at the AlN/GNP interfaces. However, the κ magnitude could be kept larger when diminishing the GNP fraction.

### 5.4. Fabrication process of substrates grading the electrical field

**Figure 7.**
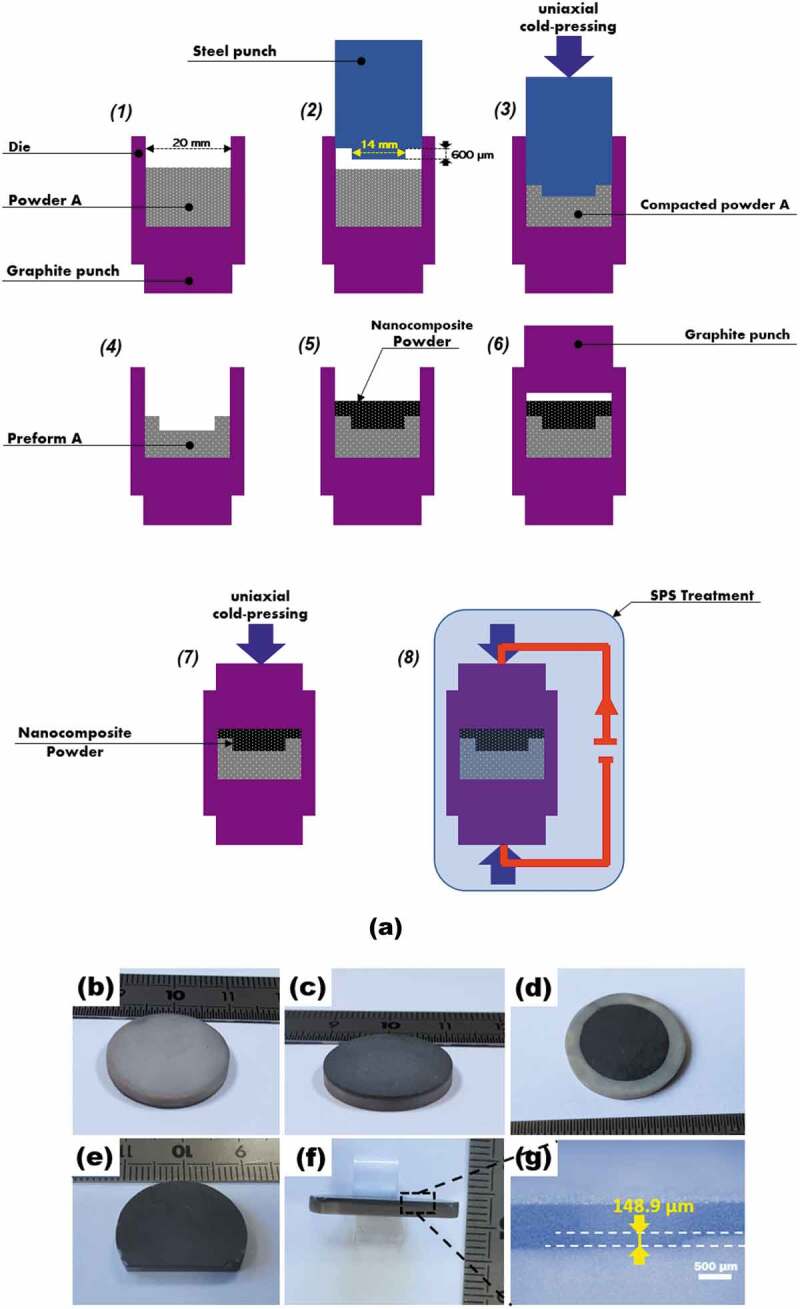
(a) Schematic illustrating the main steps enabling the three-dimensional integration of the nanocomposite within the material A. *(1) – (4)* Formation of the preform a with an imprinted cylindrical cavity having 14 mm in diameter and 600 µm in depth. *(5) – (7)* Formation of the nanocomposite that fills the cavity and covers the surface of the material A. *(8)* SPS sintering of both compacted materials a and nanocomposite in N_2_ atmosphere at 1800°C under 50 MPa for 10 min. (b and c) the respective bottom (material A) and top (nanocomposite) of the novel substrate upon the SPS processing. (d) the obtained novel substrate after removing the nanocomposite surplus covering the surface of the material A. (e and f) a cross section introduced by polishing on another novel substrate. This cross section served to investigate the interface material A/nanocomposite and to assess the dept of the nanocomposite volume into the material A. (g) Optical micrograph of such corner of such cross section allowing to experimentally measure this depth.

Finally, we developed a practical process enabling three-dimensionally integrating the elaborated nanocomposite within the material A in effort to realize the novel substrate (Figure 1). This process includes simple fabrication steps (Figure 7a) which are carried out as flows: the powder (material A) was first loaded into a 20 mm – diameter graphite die *(1)*, and a 20 mm – diameter steel punch, endowed with a cylindrical imprint (14 mm in diameter and 600 µm in height) at only one end, was then introduced in the die *(2)*. This arrangement was then uniaxially cold-pressed *(3)* with the aim of compacting the powder A and introducing an imprint cavity (14 mm in diameter and 600 µm in depth) into the resulting preform A.

Note that the dimensions of imprint of the steel punch were determined on the basis of the shrinking measurements assessed separately for materials A and B upon SPS sintering. The aim here is to obtain a substrate integrating a cylindrical volume containing the nanocomposite with a depth value the closest possible to that used in the numerical assessment of the concept, *i.e*., 150 µm (Figure 2b).

Secondly, the steel piston was removed leaving in the die only the preform A, wherein the cylindrical cavity was imprinted *(4)*. The nanocomposite powder was then poured on the preform A so as to fill the cavity and, even, cover the entire surface of this preform A *(5)*.

Finally, the die was closed by a second graphite punch *(6)* and uniaxially cold-pressed (7) before being treated in SPS under the same abovementioned conditions *(8)*. Here, it is worth noting that the SPS treatment allows to concomitantly sinter both preforms (A and nanocomposite) and joining them to each other to fabricate the novel substrate.

Figure 7 presents the obtained substrate after being polished to remove the graphite foil used during SPS sintering. Its rear and upper sides comprising material A (bright) and nanocomposite (dark) are depicted in Figures 7 b and c, respectively. It was then progressively polished on its upper side (nanocomposite) so as to remove the material excess and to keep the nanocomposite material only into the imprint cavity in order to ultimately achieve the novel substrate (Figure 7d). Optical microscopy observations indicated an excellent bonding between the materials A and nanocomposite with the absence of cracks at the interfaces between them, which reflects very low thermomechanical stresses at these interfaces, on the one hand, and emphases the relevance of the SPS cycle selected for consolidating both material A and nanocomposite, and establishing reliable three-dimensional interfaces between them, on the other hand.

Upon SPS processing, another novel substrate underwent this time a progressive polishing on its sidewall till the introduction of a cross-section exhibiting the thicknesses of both material layers (material A and nanocomposite) as shown in Figures 7 e and F. The observation of such cross-section allowed hence to assess the depth of the nanocomposite into the material A which was found to be about 148.9 µm (Figure 7g). That indicates a significant material shrinkage after sintering (about 450 µm) and, in particular, points out an excellent agreement with the dimensioned depth value used in the simulation assessment (150 µm), thereby testifying to the relevance of the approach taken here.

### 5.5. Performances of the developed substrates

**Figure 8.**
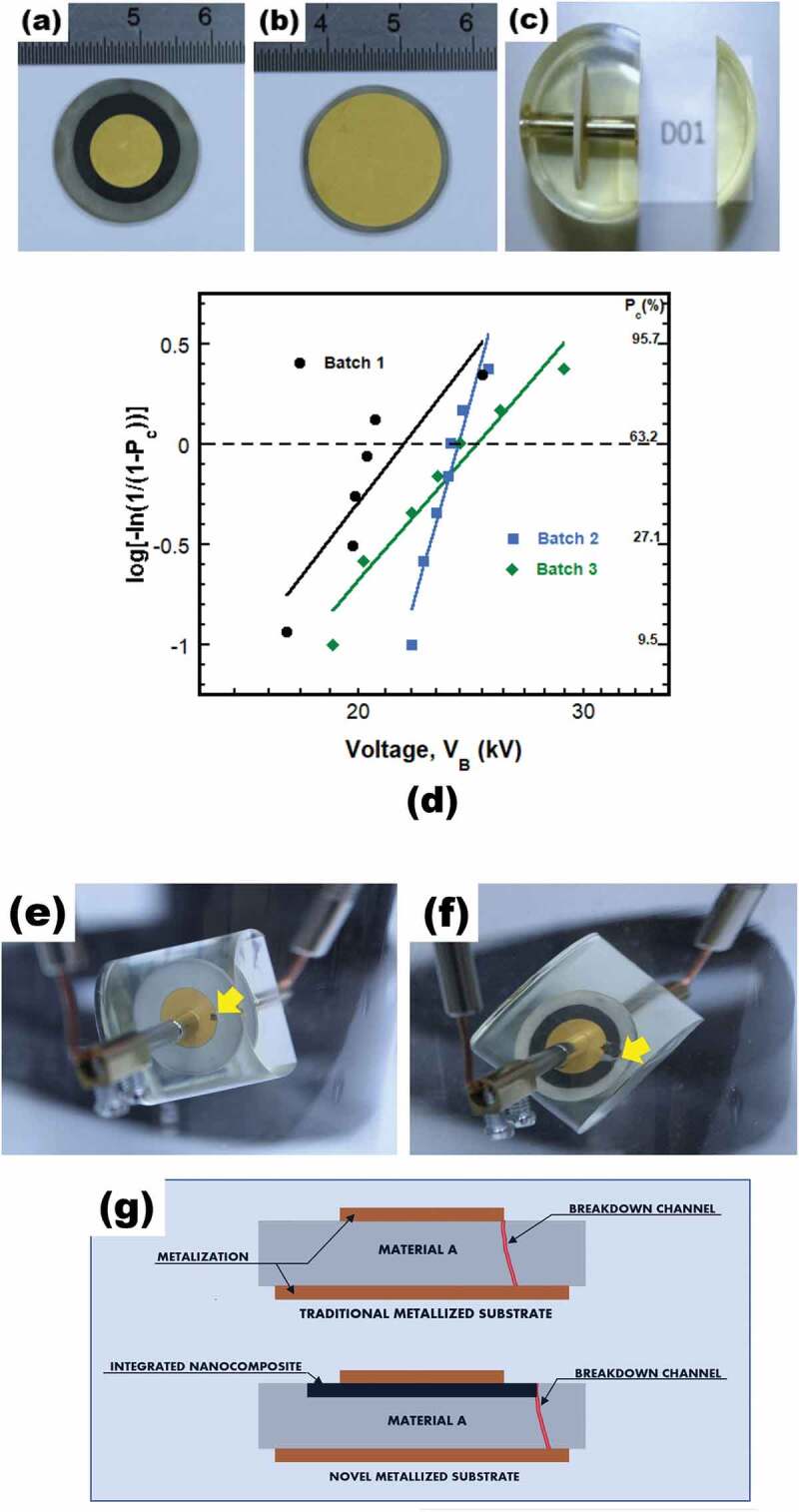
Metallization of the novel substrate achieved by depositing 150 nm thick – golden electrodes on its (a) top side (electrode with a diameter 10 mm) and (b) bottom side (electrode with a diameter 18 mm). (c) Cylindrical resin block, surrounding the assembly of metalized substrate maintained between two magnetic rods, cut to allow electrical connections. (d) Weibull plots of the voltage values obtained upon breakdown testing (AC rms −50 Hz) of batches 1, 2 and 3. (e-f) Images of tested prototype samples comprising (e) traditional and (f) novel metallized substrates showing the localization of the impact areas upon the breakdown testing. (g) Schematic illustrating the shifting of the breakdown channel from the border of the upper electrode (traditional substrate) to that of the integrated nanocomposite (novel substrate) before crossing the substrate towards the lower electrode.

In order to perform breakdown tests on the developed substrate, enabling an experimental assessment of its dielectric strength, concentric 150 nm thick – golden electrodes were first deposited on its both faces by using cathodic sputtering. In Figure 8a, a 10 mm – diameter electrode laid on the upper side of the substrate containing the integrated nanocomposite is shown, while Figure 8b depicts an 18 mm – diameter electrode layer that was deposited on its lower side.

Second, a pair of magnetic rods was used as a mean for electrically connecting the deposited electrodes. To do this, an end of each rod was brought into contact with the metalized substrate at the center of its sides, so that both rods magnetically maintain it between them. The obtained assembly was thereafter encapsulated under vacuum using transparent epoxy resin (Buheler, Epothin 2). Upon resin reticulation, the cylindrical resin block was cut so as to allow access to the second end of each magnetic rods (Figure 8c) in order to electrically connect the achieved encapsulated substrate prototype during the breakdown testing.

It is noteworthy that we realized two batches of seven similar prototype samples each from the developed substrates with the integrated materials B and C (Table 2) including fractions of 2.5 and 1.25 vol% GNP, respectively, which emphases here the very high degree of reproductivity of novel substrates using our approach.

**Table 2.** The average breakdown voltage (V_b (Average)_), and the scale (α _(pc = 63.2%)_) and shape (*β*) parameters obtained for the three batches subjected to the breakdown testing. For each batch, provided are the number of the prototype samples, the corresponding substrate and the constituent materials of the latter.

Elaborated substrates				Prototype samples			
Batch	Type substrate	Constituent materials of the substrate	Fraction of GNP incorporated in the integrated material	Number of tested samples	V_B (average)_	*α*_63.2%_	*β*
Batch 1	Traditional	Material A	-	7	20.53	21.42	8.25
Batch 2	Novel	Material B integrated in Material A	2.5 vol.%	7	23.43	23.9	22.58
Batch 3	Novel	Material C integrated in Material A	1.25 vol.%	7	23.3	24.66	7.35

To ease comparison, a third batch of seven prototype samples was prepared as well, but, this time, from traditional substrates made of only the material A (Table 2), *i.e*., without the integrated nanocomposite.

Finally, all prototype samples of the three batches were separately subjected to breakdown testing. The sample was connected and immersed in an insulating liquid (Galden HT55) within a cell designed for such testing at room temperature. A sinusoidal voltage (at 50 Hz), provided by a high voltage power supply (France Transfo, V_max_ = 100 kV), was then applied and ramped up at 1.5 kV/s. The detection of the breakdown voltage V_B_ value is performed once the electric current circulating in the circuit increases up to 5 mA with a current switching-off time of about 20 ms. The experimental breakdown data were thereafter processed using Weibull statistical distribution. Figure 8d depicts Weibull plots giving the evolution of the cumulative probability P_c_ of the breakdown as a function of V_B_ for the three sample batches. They reveal that the prototype samples comprising the novel substrates (batches 2 and 3) present larger average (V_B (Average)_) and scale parameter (α for P_c_ = 63.2%) values in comparison with the ones (batch 1) corresponding to the traditional substrate (Table 2), which reflects that the electrical constrain was mitigated at the triple point as a result of the larger σ${\;}_{⊥}$ within the integrated zones comprising the materials B and C.

Compared to the material B, the material C seems to bring a larger dielectric strength for the insulating packaging as indicated by its higher α value. It already makes V_B_ larger by 15% than that provided by the traditional substrate, and further V_B_ improvement could be attainable for lower σ${\;}_{⊥}$ by optimizing the GNP fraction within the nanocomposite. Note that the batch 2 seems to present a dispersion of V_B_ values lower than that for both other batches as indicated by its much higher shape parameter *β*.

More importantly, the location of the area degraded in the traditional substrate under the breakdown effect differs from that in the novel one. Effectively, a black mark was observed at the edge of the upper electrode of the traditional substrate as indicated on Figure 8e, while a radial black mark going from the outer edge of the integrated nanocomposite (materials B or C) to the edge of the metallization was visible on the novel substrate (Figure 8f) where such impact appeared to be surrounded by bright area as a consequence of a local resin delamination. These observations evidence that the breakdown came about for the traditional substrate under the effect of a heightened electric field at the triple point, whereas it occurred in the case of the novel substrates at the periphery of the integrated zone and created then an electrical current path joining the electrode through the nanocomposite. It suggests otherwise that the breakdown channel was shifted from the border of the upper electrode (traditional substrate) to that of the integrated nanocomposite (novel substrate) before crossing the substrate towards the lower electrode as schematized in Figure 8g.

Such shifting of the electrical field pick resulted in improving the breakdown voltage V_B_ in the prototype comprising the novel substrates, in agreement with the simulation results that furthermore revealed the potential of the latter to achieve much higher V_B_, which hence supports the merits of the concept proposed here.

## 6. Conclusions

To summarize, we demonstrate here a new way towards improving the breakdown voltage V_B_ of the insulating system of power modules by developing a novel concept of the substrate based on locally amending the properties of the traditional ceramic one. The novel substrate was fabricated by integrating, right underneath the metallization, a nanocomposite within AlN-based ceramic (material A) employing an effective process. This process, that comprises simple steps, enabled elaborating both the material A and the nanocomposite, concomitantly with joining them to each other to realize the novel substrate, thereby making significant time and energy savings. The material A displayed very low σ and high k. The nanocomposite, comprising the added GNP, was revealed to possess a controllable electrical anisotropy. For 1.25%vol GNP, it indeed showed an anisotropic electrical behavior typified by capacitive and resistive effects in the directions perpendicular and parallel to the applied pressing axis, respectively, resulting in a higher σ${\;}_{⊥}$ compared with the conductivity of the material A, and σ_*∥*_ that is nearly similar to the latter. This behavior is likely suitable for the electric field grading concomitantly with the electrical isolation function performed by the substrate. At such GNP fraction, the nanocomposite displayed a marked σ anisotropy, with (σ${\;}_{⊥}$ /σ_*∥*_) = 10^6^ (for 1.25% vol GNP) which is the highest anisotropy ratio obtained so far for the AlN-based ceramics to our knowledge.

The novel substrate presented a full densification and an excellent bonding between the materials A and nanocomposite with the absence of cracks at the interfaces between them, which testifies for the relevance of this process to achieve reliable substrates.

Electrically, it enabled shifting the breakdown channel from the border of the upper electrode (traditional substrate) to that of the integrated nanocomposite (novel substrate) before crossing the substrate towards the lower electrode, thereby leading to a larger breakdown voltage V_B_ of the novel substrate as compared to the traditional substrate. This result corroborates the numerical simulations that otherwise revealed the potential of such novel substrate in attaining further improvement in V_B_ and, subsequently, withstanding higher voltages, that could be attainable by optimizing σ${\;}_{⊥}$ through the adjustment of the GNP fraction.

We believe that the approach taken here paves the way to manufacturing multifunctional composite substrates sought not only for high power modules but also for many other potential applications.
